# An exploration of possible future non-pharmacological multidisciplinary treatments to potentiate withdrawal strategy and recovery from medication overuse headache (MOH): a narrative review

**DOI:** 10.22514/jofph.2026.032

**Published:** 2026-05-12

**Authors:** Marco Balordi, Nicholas Diani, Mattia Sinatra, Daniele Lovattini, Gianluca Rossetto, Antonio Morandi, Angelo Bellinvia, Matteo Castaldo, Alessandro Viganò

**Affiliations:** ^1^Department of Biomedical Sciences, Humanitas University, 20072 Pieve Emanuele, Italy; ^2^IRCCS Fondazione Don Gnocchi ETS, 20148 Milan, Italy; ^3^Department of Biomedical and Clinical Sciences, University of Milan, 20122 Milan, Italy; ^4^Healthcare Innovation Technology Lab, IRCCS San Camillo Hospital, 30126 Venice, Italy; ^5^Ayurvedic Point, School of Ayurvedic Medicine, 20149 Milan, Italy; ^6^Headache Centre, Department of Medicine and Surgery, University of Parma, 43126 Parma, Italy; ^7^Clinical Psychophysiology and Clinical Neuropsychology Labs, University of Parma, 43125 Parma, Italy; ^8^Neurology Unit, Humanitas Gavazzeni Hospital, 24125 Bergamo, Italy

**Keywords:** Medication overuse headache (MOH), Non-pharmacological interventions, Multidisciplinary approach, Patient education, Withdrawal

## Abstract

Medication Overuse Headache (MOH) is a secondary headache resulting from the sustained overuse of acute headache medications, and represents a clinical challenge for its complex pathophysiology and high relapse rate. This narrative review examines non-pharmacological strategies for the management of MOH, and explores the potential role of a multidisciplinary approach in improving patient outcomes. The available evidence on patient education, psychotherapy, behavioral interventions, physical therapy, and neuromodulation techniques was reviewed, along with other non-pharmacological approaches, such as dietary modifications, Ayurveda, and acupuncture. These interventions may help in targeting multiple dimensions of MOH pathophysiology, and could be tailored to individual patient needs. Integrating multimodal non-pharmacological interventions with medication withdrawal and, when appropriate, bridging therapies appears promising in improving outcomes and reducing the overall burden of MOH. However, further research is required to identify the optimal combination and duration of multidisciplinary interventions.

## 1. Introduction

Medication overuse headache (MOH) is a secondary form of headache that superimposes a chronic primary headache [1]. Specifically, MOH is diagnosed in patients experiencing ≥15 headache days per month and using an excessive number of symptomatic headache medications for more than 3 months [2]. Far from being a rare condition, MOH accounts for approximately half of all visits to specialized headache centers and affects over 70% of all patients with chronic headaches [3]. However, MOH prevalence in the general population is significantly lower, ranging from 0.5% to 7.2% across different countries [4]. This discrepancy reinforces the idea that MOH and the overuse of acute medication represent two clinical hallmarks of a more disabling headache, heralding poor response to treatments [5]. MOH is directly related to both the type and quantity of the overused acute medications. The threshold for “overuse” is defined as ≥15 days per month for nonsteroidal anti-inflammatory drugs (NSAIDs), and ≥10 days per month for other classes of medications including triptans, combination analgesics, opioids, and ergot derivatives [4]. While it seems quite robust that opioids and barbiturates have a higher chance of inducing MOH, the role of triptans in causing MOH is still debated, although their threshold remains precautionary lower than NSAIDs [6].

### 1.1 Mechanisms and factors favoring MOH

Beyond the type of acute medication used, several additional risk factors are known to influence the development of MOH. It most commonly occurs in patients with migraine as the underlying headache disorder, followed by tension-type headache (TTH), post-traumatic headache, and new daily persistent headache [7, 8]. In contrast, MOH development in patients with pure cluster headache remains controversial. Moreover, female sex, low education, economic disadvantage, and smoking are all associated with a higher risk of MOH [9].

Among the modifiable risk factors, metabolic syndrome, obesity, and physical inactivity are strongly linked to MOH. Whereas the first two conditions are generally associated with chronic migraine (CM), physical inactivity (defined as <3 hours of vigorous physical activity per week) is specifically associated with the risk of MOH [10].

Musculoskeletal pain and other co-existing painful conditions represent additional risk factors for MOH. The relationship between multiple pain disorders is so closely intertwined that recently ten conditions (*i.e.*, musculoskeletal pain, temporomandibular disorder, fibromyalgia, irritable bowel syndrome, vulvodynia, myalgic encephalomyelitis/chronic fatigue syndrome, interstitial cystitis/painful bladder syndrome, endometriosis, and pelvic floor pain) are now grouped under the term chronic overlapping pain conditions (COPCs) [10, 11].

Moreover, psychiatric comorbidities are more prevalent in MOH. Most notably, depression and anxiety have been consistently reported [12], and in MOH patients with multiple therapeutic failures, also bipolar disorder is quite common [13]. Furthermore, MOH patients tend to show different personality traits compared with patients without MOH [14].

This result is also in line with new evidence showing that both childhood emotional and physical trauma are strong determinants of the future development of MOH. In particular, when childhood trauma is associated with the presence of alexithymia, the risk of developing a somatization disorder increases [15].

The possible overlap between patients with MOH and those with substance use disorder remains controversial. However, a seminal neuroimaging study has shown that the orbitofrontal cortex remains metabolically hypoactive even after withdrawal therapy, whereas other brain regions previously affected by MOH return to a metabolic activity comparable to that of healthy controls [16].

A recent study suggests that CM patients could be divided into two categories. On one side, “pure” CM with less comorbidities and risk factors, who have a low risk of developing MOH and who can, in general, experience transient medication overuse. On the other side, CM patients with multiple MOH risk factors, who are more likely to develop MOH early in the course of migraine chronicity or to relapse after withdrawal. In “pure” CM patients without prior history of MOH, the annual incidence of MOH is approximately 17% per year, increasing to 33% in those unresponsive to preventive treatments [17]. However, in patients with a previous history of MOH, relapse rates increase up to 66–78% in the following years [18, 19].

Taken together, these data explain why the management of MOH has always represented a complex issue in the headache field. For many years, it has been debated whether a standardized detoxification strategy is necessary to interrupt medication overuse (MO) or whether effective headache prevention alone may be sufficient to avoid the development of MOH. This debate has become particularly relevant in recent years with the introduction of new preventive medications, which have reinforced the idea of avoiding formal detoxification [20].

### 1.2 Chronification mechanisms in MOH patients

While transient MO without progression to MOH can also occur in episodic migraine (EM) patients [21], the development of MOH is typically accompanied by a progressive increase in headache frequency, and by specific modifications of neuronal excitability at peripheral and central level of the nervous system. Notably, emerging evidence suggests that some of these modifications may be partially reversible in the presence of protective factors [17].

Among the mechanisms implicated, central sensitization is probably one of the most important processes involved in MOH, as demonstrated by neurophysiological studies in humans [22] and mice models [23, 24]. In humans, functional magnetic resonance imaging (fMRI) studies reported findings consistent with central sensitization, showing stronger pain-induced responses within the pain neuromatrix and in several brain regions, including the motor cortex, superior temporal sulcus, premotor cortex, supplementary motor area, lingual gyrus, dorsomedial prefrontal cortex, anterior cingulate cortex, and primary somatosensory cortex [25]. Compared with CM without MOH, those with comorbid MOH exhibit more severe functional and structural alterations [25, 26]. Encouragingly, these alterations seem to revert following recovery from MOH [27].

### 1.3 Evidence for multidisciplinary integrated pharmacological and non-pharmacological treatment

Due to the complexity of MOH presentations, a multidisciplinary, integrated approach is increasingly viewed as a valuable strategy for management [28]. While not all MOH patients may require such an approach, those with high relapse risk, previous treatment failures, severe clinical burden, or psychiatric comorbidities are likely to benefit from a combined therapy [28]. To date, however, several elements of this approach remain insufficiently defined, including the specific components of the intervention, involved specialties, and optimal composition of the multidisciplinary team. In most studies, teams comprise neurologists, psychologists, physiotherapists, and headache nurses, although some evidence also supports the inclusion of occupational therapists, health educators, and aerobic exercise trainers [29]. Another key consideration is the setting in which the intervention is delivered. Current evidence suggests that outpatient or day-hospital models offer comparable clinical effectiveness [30], while also providing greater practicality and cost-efficiency [31]. An important exception to an outpatient setting arises when patients present significant systemic comorbidities, require withdrawal from drugs such as opioids or barbiturates, or have previously failed outpatient detoxification attempts [32]. In addition, with regard to the optimal duration of intervention, no consensus has been reached, with available evidence ranging from 180 minutes to 96 hours [28].

The major advantage of non-pharmacological interventions lies in their applicability even in presence of multiple comorbidities. In fact, they may be especially beneficial for patients with conditions such as anxiety, depression, asthma, obesity, or metabolic syndrome. These comorbidities not only predict poorer outcomes [33], but represent *per se* an impediment to use some pharmacological preventive therapies—either due to contraindications or tolerability issues. For instance, beta-blockers are contraindicated in asthma, valproate is discouraged in obesity, and topiramate should be avoided in patients with suicide ideation.

Despite growing interest, multidisciplinary approaches for MOH have not been systematically compared to other strategies of MOH management, with the exception of a few studies [31, 34]. Nonetheless, observational studies found responder rates above 50%, ranging from 43% to 63% [29, 35, 36]. An open-label study compared gradual detoxification to abrupt withdrawal combined with multidisciplinary education. Both groups achieved a ≥80% success rate, however the multidisciplinary education group required fewer staff resources and needed fewer prophylactic drugs after detoxification [34]. A visual representation of several non-pharmacological interventions used in the multidisciplinary management of MOH is provided in Fig. 1, while Table 1 (Ref. [34, 35, 37, 38, 39, 40, 41, 42, 43, 44, 45, 46, 47]) summarizes and compares the main characteristics of the included studies.

**Fig. 1.** Visual representation of several non-pharmacological approaches for the multidisciplinary management of MOH. Created in BioRender. Balordi M. (2026) https://BioRender.com/tm0uc2b. EMDR: eye movement desensitization and reprocessing; PNE: pain neuroscience education.

**Table 1.** Cited articles for each non-pharmacological intervention.

Study	Sample	Study type	Number of disciplines	Disciplines	Outcomes	% of MOH patients	Notes
Munksgaard *et al*. [34] 2021	98	Open-label	4	Neurologist, Physiotherapist, Psychologist, Nurse	Multiple Outcomes	100%	Resistant MOH
Zeeberg *et al*. [37] 2005	336	Retrospective observational—single arm	7	Neurologist, Physiotherapist, Psychologist, Nurse, Psychiatricist, Dentist, Gynecologist	Multiple Outcomes	80%	Non comparison between patients with and without MOH
Jensen *et al*. [38] 2010	1326	Retrospective observational—single arm	4	Neurologist, Physiotherapist, Psychologist, Nurse	Reduction in MHDs	25.5%	
Lemstra *et al*. [39] 2002	84	RCT	3	Neurologist, Physiotherapist, Psychologist	Multiple Outcomes	Not clear	No difference between multidisciplinary and standard care group
Harpole *et al*. [40] 2003	54	Prospective observational	2	Neurologist, Psychologist	Multiple Outcomes	20.4%	
Maizels *et al*. [42] 2003	264	Prospective observational	3	Family physician, nurse practitioner, clinic coordinator	Reduction of severe headache days	39.7%	Possible bias due to an increase in acute medication cost
Gunreben-Stempfle *et al*. [41] 2009	42	Prospective observational	3	Neurologist, Physiotherapist, Psychologist	Number of >50% responder	Not clear	Comparison with historical cohort
Lake *et al*. [47] 2009	267	Retrospective observational—single arm	6	Neurologist, Psychologist, Anesthesiologist, Physical Therapist, Occupational Therapist, Specialized Nurse	Multiple Outcomes	59.17%	
Wallasch *et al*. [35] 2012	201	Prospective, observational study	3	Neurologist, Physiotherapist, Psychologist	Number of >50% responder	12.4%	Plus Hospitalization for 5 days
Rothrock *et al*. [43] 2009	100	RCT	2	Neurologist, Education provider	MIDAS score	Not clear	*vs.* standard care
Gaul *et al*. [44] 2011	295	Prospective observational study	4	Neurologist, Education provider, Psychologist, Nurse	Reduction in MHDs	56%	
Magnusson *et al*. [45] 2004	127	RCT	4	Neurologist, Physiotherapist, Psychologist, Nurse	QoL Scales	43%	
Matchar *et al*. [46] 2008	614	RCT	2	Neurologist, Nurse	MIDAS score	Not clear	Educational intervention

*MOH: medication overuse headache; RCT: randomized controlled trial; MIDAS: Migraine Disability Assessment Score Questionnaire; QoL: Quality of Life; MHD: month headache days.*

## 2. Methods

The present article is a narrative review of the literature on non-pharmacological and multidisciplinary interventions for the management of MOH. A narrative approach was selected instead of a systematic review or meta-analysis because of the substantial heterogeneity of non-pharmacological treatments and the wide variability of interventions in terms of intensity, number, and frequency of sessions. In addition, many non-pharmacological trials on CM or chronic TTH, often exploratory in nature, do not clearly report whether patients with MOH were included or excluded. Given that a large proportion of patients with CM also meet criteria for MOH, non-pharmacological studies on CM were included unless it was explicitly stated that only patients with CM were enrolled or that other primary headache disorders were excluded.

The research team conducted a structured non-systematic literature search through PubMed/MEDLINE and Google Scholar databases. The search was conducted in July 2024 and updated in May 2025. Keywords included combinations of terms such as “medication overuse headache”, “MOH”, “non-pharmacological treatment”, “multidisciplinary treatment”, “patient education”, “psychotherapy”, “physical therapy”, “neuromodulation”, and “complementary medicine”.

We included peer-reviewed articles in English, published from 2000 up to May 2025, including selected earlier works of historical or clinical relevance. Eligible studies involved adult populations and addressed non-pharmacological or multidisciplinary interventions relevant to MOH. The research excluded studies focusing exclusively on pharmacological approaches, pediatric populations, case reports, and non-peer-reviewed publications.

Finally, findings were synthesized thematically and presented according to intervention type. Given the heterogeneity of methodologies, no formal quality assessment or quantitative synthesis was conducted.

## 3. Guidelines and recommended strategies to manage MOH

### 3.1 Withdrawal and patients’ education

To date, the mainstay of MOH management remains the discontinuation of the overused medication through withdrawal therapy that should be implemented even when other therapeutic approaches are not possible, although recent evidence showed that combing it with preventive therapy provides a better outcome [48, 49, 50]. This may indeed be complemented by bridging analgesics and/or prophylactic agents, if deemed appropriate. Nevertheless, further research is warranted to optimize this strategy.

Withdrawal protocols vary also depending on the type of overused drug. Inpatient management is recommended for patients overusing opioids, benzodiazepines, or barbiturates, while outpatient detoxification is generally considered sufficient for other medication classes [51]. The choice between abrupt withdrawal and gradual tapering remains a topic of debate [52]. Gradual tapering is advised for barbiturates and opioids to mitigate withdrawal symptoms, whereas for other drugs, abrupt cessation appears more effective than gradual reduction [3]. Importantly, evidence suggests that structured detoxification program, when paired with close follow-up, is highly effective [34]. Regardless of the modality of medication interruption, sustained avoidance of medication overuse is associated with improved medium- and long-term outcomes [53, 54, 55].

### 3.2 Bridging therapy and preventive therapy

Bridging therapy with analgesics could be primarily offered to patients in the outpatient setting, to increase patient compliance and as alternative to down tapering alone. Long-lasting analgesics (*e.g.*, naproxen) are the drug-of-choice in bridging therapy for a couple of weeks. The duration of bridging therapy is based on the duration of withdrawal symptoms, lasting for up to 10 days for NSAIDs, around 4 days for triptans, and about 7 days for ergot derivatives [56]. During hospitalization or day-hospital withdrawal [57], intravenous administration of acetylsalicylic acid [58], or corticosteroids [59, 60] may be used alone or in combination with supportive measures such as hydration, antiemetics, clonidine, or benzodiazepines. Notably, corticosteroids appear to be the most controversial choice among the possible rescue medications [61].

Patient education represents a crucial component in the management of MOH [54]. Explaining, in accessible terms, what MOH is and how it develops enables patients to adopt appropriate behaviors to discontinue MO, while at the same time maintaining adherence to prescribed treatments. MOH education primarily aims to inform patients about the role of acute medications in perpetuating headaches, and to emphasize the importance of interrupting overuse in order to revert chronicity and prevent recurrence. In the past, some seminal studies have shown that, in patients with uncomplicated MOH, this “advice-only” approach can achieve detoxification rates comparable to those of more intensive inpatient or outpatient medication-based programs [50, 52]. However, this net benefit cannot be generalized to patients with complicated MOH [49].

Another critical issue in the management of MOH concerns the timing of preventive therapy initiation—whether it should be started concomitantly with withdrawal or delayed until after a period of withdrawal alone, typically lasting three months [34]. This question remains unresolved and is generally addressed on a case-by-case basis, taking into account factors such as previous failed withdrawal attempts, headache burden, and the presence of comorbidities, including psychiatric conditions [52]. Recent evidence suggests that preventive therapy may be more effective than withdrawal alone, particularly following the introduction of new migraine-specific preventive agents. However, the actual role and magnitude of the effect of these medications in MOH management remain under investigation [62, 63]. Reported rates of MOH resolution with preventive treatments ranges widely, from 29% to 88% of patients [64]. While some authors propose that these new therapies may reduce the need for, or even allow the omission of, formal withdrawal, others support the opposite approach [20, 65]. One potential confounding factor in this debate is drug dosage, which may differentially influence outcomes, as demonstrated for monoclonal antibodies administered at different doses [66]. Further studies are needed to better clarify the impact of calcitonin gene-related peptide (CGRP)-targeting monoclonal antibodies in MOH management.

### 3.3 Psychotherapy support

Due to the close relationship between the development of MOH and emotional distress, dysfunctional thoughts, psychiatric and psychological comorbidities [67], the pairing of pharmacological treatments with psychological support could help patients with coping and prevent MOH relapses [68]. The European Academy of Neurology endorsed Short-term Psychodynamic Psychotherapy (STPP) and mindfulness as potentially helpful in MOH management [68].

STPP is a Freudian-inspired psychotherapy approach that considers migraine headache as a psychosomatic disorder. It attributes the condition to the patient’s previous traumatic experiences, suffering, and aspirations, which are considered in terms of intra-psychic conflicts [69]. Mentalization—the ability to process emotional conflicts and express them through words—emerges as a key protective factor. This ability is shaped by a combination of personal and environmental factors, with higher levels of mentalization linked to more sophisticated and effective emotional regulation. In turn, this is associated with a lower risk of psychosomatic symptoms and, consequently, a reduced likelihood of migraine chronification. In a recent study, we found that in accordance with the STPP model, CM and MOH patients tend to express low or intermediate levels of mentalization, which may favor the development of chronic headache symptoms. Therapeutic STPP aims at helping the patient to elaborate past conflicts and the associated emotional burden, in order to get a resolution of both intra-psychic conflicts and consequently headache symptoms [70].

Mindfulness, defined as a focused, non-judgmental awareness of the present moment, has been investigated in migraine, but to date its real effectiveness is under investigation since recent systematic reviews have not established a clear benefit [71]. However, recent phase III trials involving 177 patients, reported that mindfulness, when added to standard care (*i.e.*, withdrawal, education, and prophylaxis), led to greater improvements in terms of headache frequency, medication intake and quality of life (QoL) [72].

## 4. Future possible therapeutic options for the non-pharmacological management of MOH

Besides the therapies and approaches discussed above, several emerging non-pharmacological interventions may hold potential in the field of migraine field and, in particular, in the management of MOH, given the complex clinical presentation of these patients. However, most of these interventions have been investigated only in recent years, with preliminary findings that are promising, but limited in number and often lacking replication. Moreover, the available evidence largely derives from studies conducted in the broader migraine population, with only a few investigations specifically targeting patients with MOH. As a result, the overall level of evidence supporting their efficacy in MOH remains low. Nevertheless, owing to their favorable safety profiles, some of these approaches warrant further investigation in future studies. In the following section, a brief outline is provided regarding non-pharmacological interventions that may represent potential adjuncts to standard therapy in the management of MOH.

### 4.1 Additional psychotherapy treatments: CBT, EMDR and other behavioral interventions (such as pain neuroscience education)

Among the most commonly used psychotherapeutic approaches, cognitive-behavioral psychotherapy (CBT) is one the most extensively investigated in the field of migraine, particularly in combination with other behavioral techniques. However, its specific role in the management of MOH is less clearly defined than for other headache conditions [73]. CBT aims to help patients process pain-related negative emotions and dysfunctional thoughts, identify triggers, modify maladaptive habits, and ultimately develop preventive coping strategies [74]. In patients with MOH, CBT combined with other behavioral interventions has been evaluated in a double-blind RCT, delivered by trained professionals during the withdrawal phase, and was associated with MOH resolution in approximately 7% more patients compared with standard therapy alone [75].

Another possible psychotherapeutic approach that may be of interest in the management of MOH is Eye Movement Desensitization and Reprocessing (EMDR). This technique is typically proposed as an adjunctive intervention within a psychotherapeutic framework, primarily for the treatment of post-traumatic stress disorder [76]. EMDR involves bilateral visual, auditory, and tactile stimulation aimed at facilitating the desensitization and reprocessing of traumatic memories. Two studies have investigated the use of EMDR in patients with migraine, without detailed characterization of patient profiles (see review [77]). The two studies differed in the modality of EMDR delivery, namely visual-only versus combined visual–tactile stimulation. The first study focused on headache-related traumatic experiences and reported that three months of EMDR were associated with reductions in both headache days and acute medication use, with sustained benefits at follow-up [77]. The second study, a larger RCT, showed that EMDR used as an add-on to standard preventive therapy led to faster and more pronounced pain relief [77].

Given the central role of patient education in the management of MOH, one might speculate that a more structured and in-depth educational approach may be more effective than brief or purely informational interventions, such as providing short counselling or written materials. Pain Neuroscience Education (PNE) may represent a valuable resource for implementation in patients with MOH. While traditional biomedical education primarily focuses on tissue damage as the cause of pain [78], PNE aims to enhance patients’ understanding of pain neurophysiology and neurobiology, pain representation, and the meaning of pain. PNE is considered a behavioral intervention that primarily targets coping strategies, which are often dysfunctional in patients with migraine and MOH [79, 80, 81]. Indeed, PNE has been shown to improve coping strategies and scores on the Central Sensitization Inventory, Pain Catastrophizing Scale, and Tampa Scale of Kinesiophobia, even in the absence of a direct effect on pain intensity [82].

Recently PNE has been implemented with promising results in several chronic pain conditions, including cancer-related pain [83, 84, 85, 86]. However, for other chronic pain entities, including fibromyalgia and headache in general, the evidence remains limited [87, 88, 89]. Recent reviews focusing on PNE in migraine have reported moderate to strong evidence of effectiveness, although uncertainties remain regarding optimal implementation parameters, including delivery modalities and the number of sessions required [90, 91]. At present, only a limited number of trials specifically targeting MOH are available. However, several studies in patients with CM, with and without MOH, are currently ongoing [92, 93]. Although the existing literature does not yet support the routine application of PNE in the management of MOH, it is noteworthy that the benefits of PNE appear to be enhanced when it is combined with other non-pharmacological interventions, yielding better outcomes than single intervention alone (*e.g.*, physical therapy) [94]. Overall, these findings may suggest that PNE could be worthy of further investigation in future MOH-focused studies.

### 4.2 Manual therapy, exercise, and dry needling

Physiotherapy (PT) offers a broad spectrum of non-pharmacological interventions for patients with migraine and MOH, including, among others, exercise programs, manual therapy (MT), and dry needling (DN) [95]. Unfortunately, PT has only recently been studied in the context of MOH with a single study by Trager *et al*. [96]. This study analyzed data from the USTriNetX network (covering over 124 million individuals) and found that patients who received MT were less likely to develop MOH over the following 2-years, compared with those who did not receive MT. The main rationale for PT in migraine, and MOH in particular, lies in its ability to address musculoskeletal disorders (*e.g.*, neck pain) contributing to headache symptoms [95, 97]. PT interventions in migraine may also include postural rehabilitation, balance training, and vestibular exercises, while encouraging active patient engagement and promoting lifestyle modifications [98]. Although the overall quality of evidence is still low, a recent meta-analysis showed that specific combinations of MT, exercise, and electrical stimulation can be effective in treating TTH [99]. In migraine, more complex PT strategies—such as occipital transcutaneous electrical stimulation, acupressure, osteopathic MT, soft tissue mobilization, facial proprioceptive neuromuscular facilitation, and aerobic exercise—have also demonstrated some efficacy [95].

For chronic headaches, MT hands-on techniques (*e.g.*, massage therapy, joint mobilization and spinal manipulation) could be valuable adjunctive treatments [100]. Both soft tissue and articulatory MT have shown benefits in migraines [101]. In particular, spinal manipulative therapy has been shown to significantly reduce headache frequency and intensity in chronic headache, with effects comparable to those of propranolol, topiramate, and amitriptyline [100]. However, uncertainties remain regarding standardized techniques to be used, the optimal number of sessions, and the appropriate duration of treatment.

Aerobic exercise regimens are also included in the physiotherapeutic armamentarium for migraine management [98]. Although in some cases physical activity may trigger migraine attacks or exacerbate chronic pain, regular exercise appears to reduce both migraine frequency and pain perception over time [102, 103]. Clinical guidelines from the French, Danish, and American Headache Societies support the inclusion of exercise as part of migraine management [104, 105, 106]. Accordingly, aerobic exercise and physical activity combined with healthy lifestyle habits are classified as grade B recommendations for migraine management [107]. In particular, strength training has been identified as the most effective exercise modality for both CM and chronic TTH [108, 109]. Moreover, both high- and moderate-intensity aerobic exercise have shown efficacy comparable to that of topiramate and amitriptyline in migraine management [110]. Nevertheless, as in the case of MT, the application of exercise-based therapy lacks standardization, and further research is required to define optimal protocols and to account for variables such as patient preferences, fitness levels, and psychological factors [111].

Dry needling (DN) is a puncture technique based on inserting needles into painful areas, such as myofascial trigger points, sites whose stimulation is able to elicit local and referred pain [112, 113]. However, evidence on the efficacy of DN for headache remains very limited, due to heterogeneous headache types or lack of superiority over controls.

### 4.3 Non-invasive neuro-modulation

Non-invasive neuromodulation has been investigated over the past decade in the context of migraine and CM. The rationale for its use lies in the potential to reverse maladaptive neuroplastic changes underlying central sensitization and the chronification process [114]. However, with the exception of single pulse transcranic magnetic stimulation (sTMS) and peripheral transcutaneous electrical nerve stimulation (TENS) for the prevention of migraine with aura [115, 116], current evidence provides limited support for the routine use of neuromodulation in CM and, in particular, in MOH.

Peripheral TENS has demonstrated a good level of evidence in episodic migraine [115], whereas only one open-label study has evaluated its use in patients with CM and concomitant MOH [117]. Despite the inherent limitations of the open-label design, 34.8% of patients achieved both a >50% response from baseline and discontinuation of MO.

Evidence regarding transcranial direct current stimulation (tDCS) remains heterogeneous. A recent review found that tDCS may reduce migraine frequency, pain intensity, and acute medication intake, without identifying a clear superiority between anodal and cathodal stimulation protocols [118]. Few studies have specifically addressed MOH. De Icco *et al*. [119] (2021) conducted a double-blind RCT with anodal tDCS applied for 5 days, targeting one of the primary motor cortices (according to pain) during inpatient MO withdrawal. Clinical follow-ups at 1 and 6 months showed a significant clinical benefit in the active stimulation group. However, no concomitant reduction in acute medication intake was observed [119]. In contrast, a three-arm RCT involving 135 patients with MOH compared anodal, cathodal, and sham tDCS, with a 1-year follow-up, yielding overall negative results. The tDCS was delivered for five consecutive days during drug withdrawal on the right primary motor cortex, using the same stimulation parameters of the study by De Icco *et al*. [119]. Despite the overall reduction of headache days over the 12-month period, the proportion of >50% responders did not differ significantly between groups (64.1% for anodal, 60.0% for cathodal, and 46.3% for sham) [120].

Cathodal occipital tDCS was found to be more effective than anodal dorsolateral prefrontal cortex (DLPFC) and sham stimulation in patients with MOH at two weeks post-intervention, particularly in reducing acute medication consumption [121].

A further study explored the concomitant stimulation of two cortical targets in a cohort of patients with CM and MOH who were resistant to multiple preventive treatment lines and presented with major psychiatric comorbidities [13]. Anodal stimulation was applied to the right instead of left DLPFC (a montage previously used in other studies to reduce craving symptoms [122]), while cathodal stimulation targeted the occipital cortex. All patients experienced significant reductions in headache and migraine days per month, as well as acute medication use. Notably, greater reductions in headache frequency were accompanied by improvements in psychiatric symptoms [13].

Transcranial magnetic stimulation (TMS), both single pulse (sTMS) and repetitive pulse (rTMS), has also shown promise. In one study, sTMS was administered to 153 treatment-resistant patients with high-frequency CM, with or without MOH. Patients received increasing doses of sTMS over three months, up to 6 pulses three times daily. At 12-month follow-up, approximately 45% of patients achieved sustained clinical improvement, and the prevalence of MOH decreased from 52% at baseline to 8% [123].

In a more recent study focused exclusively on MOH [124], 12 patients underwent inhibitory quadripulse repetitive TMS (rTMS) applied to the occipital cortex twice weekly for one month [125]. This intervention resulted in a reduction of approximately 8 headache days per month, with patients reverting from CM to episodic migraine and showing improved habituation.

### 4.4 Complementary non-pharmacological techniques

Several complementary non-pharmacological approaches, which remain underexplored in chronic headache and MOH, may be considered as adjuncts to standard treatment strategies in MOH. Among these, dietary interventions are probably the most widely recognized. Diet is known to influence migraine through mechanisms involving metabolism, gut microbiota, and systemic inflammation [126]. However, a structured and systematic application of dietary interventions as a therapeutic strategy in MOH patients is still lacking [127]. Recent findings suggest patients with MOH more frequently present comorbid irritable bowel syndrome and that dopaminergic foods are more likely to trigger headache attacks in this population, whereas histaminergic foods appear to be more commonly associated with migraine attacks in patients without MOH [128].

In parallel, overweight and obesity have been shown to be independently associated with MOH even after multivariable adjustment for several confounding factors (*e.g.*, age, sex, and education level), with a proportional relationship between increasing body mass index (BMI) and headache burden [129]. Consequently, weight loss in obese patients has been consistently associated with reductions in headache frequency and headache-related disability [130]. Some dietary approaches may be more effective than others [131], although concerns regarding long-term safety remain [132].

Other complementary approaches, such as acupuncture and Ayurveda, may also be considered as adjunctive tools in MOH management, although evidence supporting their use and their underlying mechanisms remains limited.

Acupuncture, a traditional Chinese medicine technique employed in several neurological disorders [133], has not yet been selectively investigated in MOH. Recently, it was evaluated as an add-on treatment to topiramate in a single-blind, double-dummy RCT, demonstrating favorable outcomes compared with topiramate alone [134]. Furthermore, a recent network meta-analysis comparing acupuncture with topiramate and botulinum toxin type A (BoNT-A), found that acupuncture was not superior to BoNT-A in terms of efficacy [135]. Significant uncertainties remain regarding optimal treatment parameters for the delivery of acupuncture, including dosing, selection of target body points (among the 365 recognized locations), treatment frequency, and the ideal number of sessions [136]. Ayurveda, a traditional Indian medical system, approaches migraine management through interventions such as orally administered herbal preparations, as well as medicated oils applied nasally or used in therapeutic massage [137]. Due to the intrinsic challenges in standardizing Ayurvedic treatments, which are typically tailored to the individual patient, rigorous investigation remains difficult. However, novel research designs have been proposed to improve reproducibility in this field [138]. An important consideration in the potential use of Ayurvedic therapies is the risk of herb-drug interactions [139].

## 5. Limitations

The present article has several limitations that should be acknowledged. From a methodological perspective, this is a narrative review and, therefore, subject to inherent limitations that must be considered when interpreting its findings. These include potential selection bias in the literature search and limited reproducibility. Moreover, by their nature, narrative reviews reflect the authors’ interpretation of the available evidence, rather than a systematic and quantitative synthesis. As a consequence, reviews conducted by other authors using different methodologies may lead to partially different conclusions. From the conceptual perspective, we deliberately adopted an inclusive approach toward non-pharmacological interventions, including also trials conducted on CM population in which the exclusion of MOH patients was not explicitly stated, given the close epidemiological and clinical overlap between these conditions. We acknowledge that the joint consideration of CM and MOH populations may be debated. While this approach allowed us to explore a broader range of potential therapeutic strategies, the findings should be interpreted with caution, and further dedicated studies are required before these interventions can be routinely implemented in clinical practice.

## 6. Conclusions

Patients with MOH account for a large proportion of cases referred to headache centers, and withdrawal of the overused medication remains the mainstay of treatment. Nevertheless, relapse or treatment failure occurs in up to 50% of patients [140]. Current standard of care typically consists of withdrawal of the overused medication, brief educational interventions addressing the risks of MOH, and the initiation of pharmacological preventive therapies aimed at reducing migraine burden and limiting dropout rates [3].

Integrating different non-pharmacological approaches and combining them with pharmacological treatments may offer synergistic and more personalized therapeutic effects in patients with MOH. Such interventions should be framed within a biopsychosocial model that accounts for individual psychological factors, social context, and lifestyle habits [141, 142]. The concurrent implementation of multiple non-pharmacological strategies may represent a promising avenue to improve treatment adherence and clinical outcomes in MOH management. Further well-designed studies in this area seem both necessary and worthwhile.
